# Supplementary material for: Long-read sequencing transcriptome quantification with lr-kallisto
Source: bioRxiv. 2024 Sep 9:2024.07.19.604364. Originally published 2024 Jul 19. Preprint. [Version 2] doi: 10.1101/2024.07.19.604364 (PMC11275803; doi:10.1101/2024.07.19.604364)
Supplement: Supplementary file 1 [file media-1.pdf]

Supplementary Figures
Long-read sequencing transcriptome quantification with lr-kallisto

Rebekah K. Loving[1], **Delaney K. Sullivan**[1,2], **Fairlie Reese**[3,4], **Elisabeth Rebboah**[3,4], **Jasmine Sakr**[3,4], **Narges Rezaie**[3,4], **Heidi Y. Liang**[3,4], **Ghassan Filimban**[3,4], **Shimako Kawauchi**[3], **Conrad Oakes**[1], **Diane Trout**[1], **Brian A. Williams**[1], **Grant MacGregor**[3], **Barbara J. Wold**[1], **Ali Mortazavi**[3,4], and **Lior Pachter**[1,5]

[1]Division of Biology and Biological Engineering, California Institute of Technology, Pasadena, CA 91125
[2]UCLA-Caltech Medical Scientist Training Program, David Geffen School of Medicine, University of California, Los Angeles, Los Angeles, CA, 90095, USA
[3]Developmental and Cell Biology, University of California Irvine, Irvine, USA
[4]Center for Complex Biological Systems, University of California Irvine, Irvine, USA
[5]Department of Computing and Mathematical Sciences, California Institute of Technology, Pasadena, CA 91125

Correspondence: ***Ali Mortazavi (ali.mortazavi@uci.edu) and Lior Pachter (lpachter@caltech.edu)***

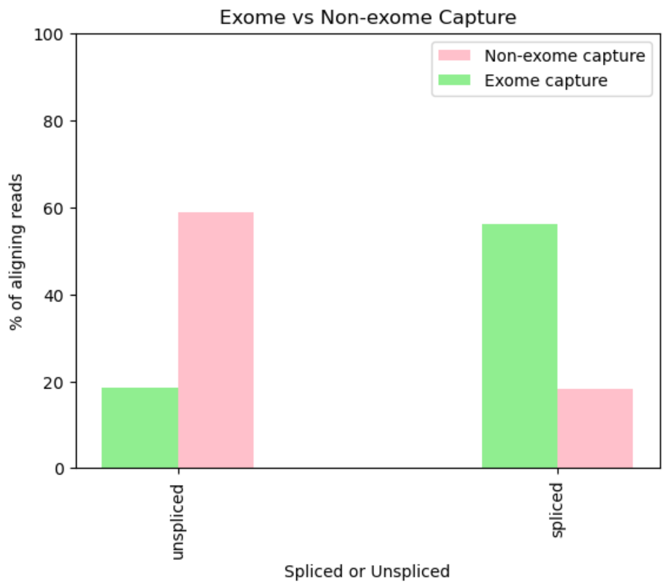

Supplementary Fig. 1a: Comparison of the percent of reads mapping as spliced vs. unspliced reads with and without exome capture.

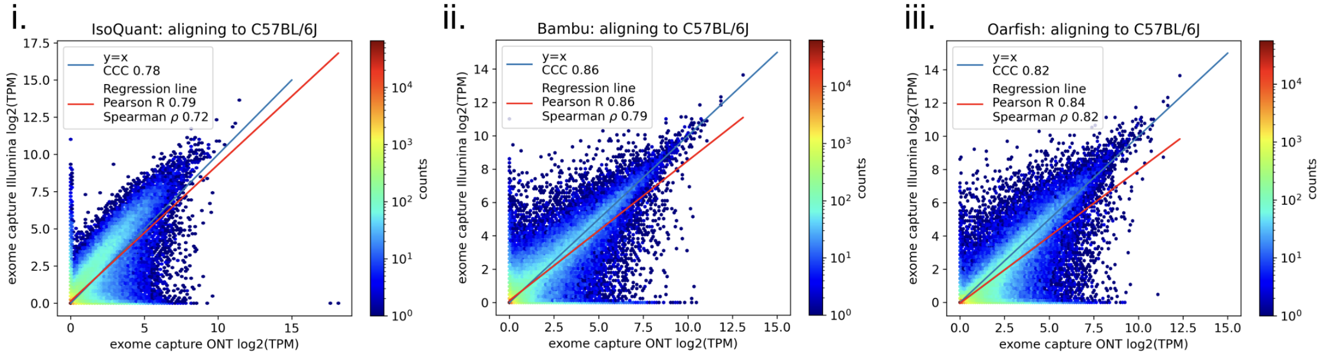

Supplementary Fig. 1b: Quantifications of the C57BL/6J exome capture samples with Bambu, IsoQuant, and Oarfish.

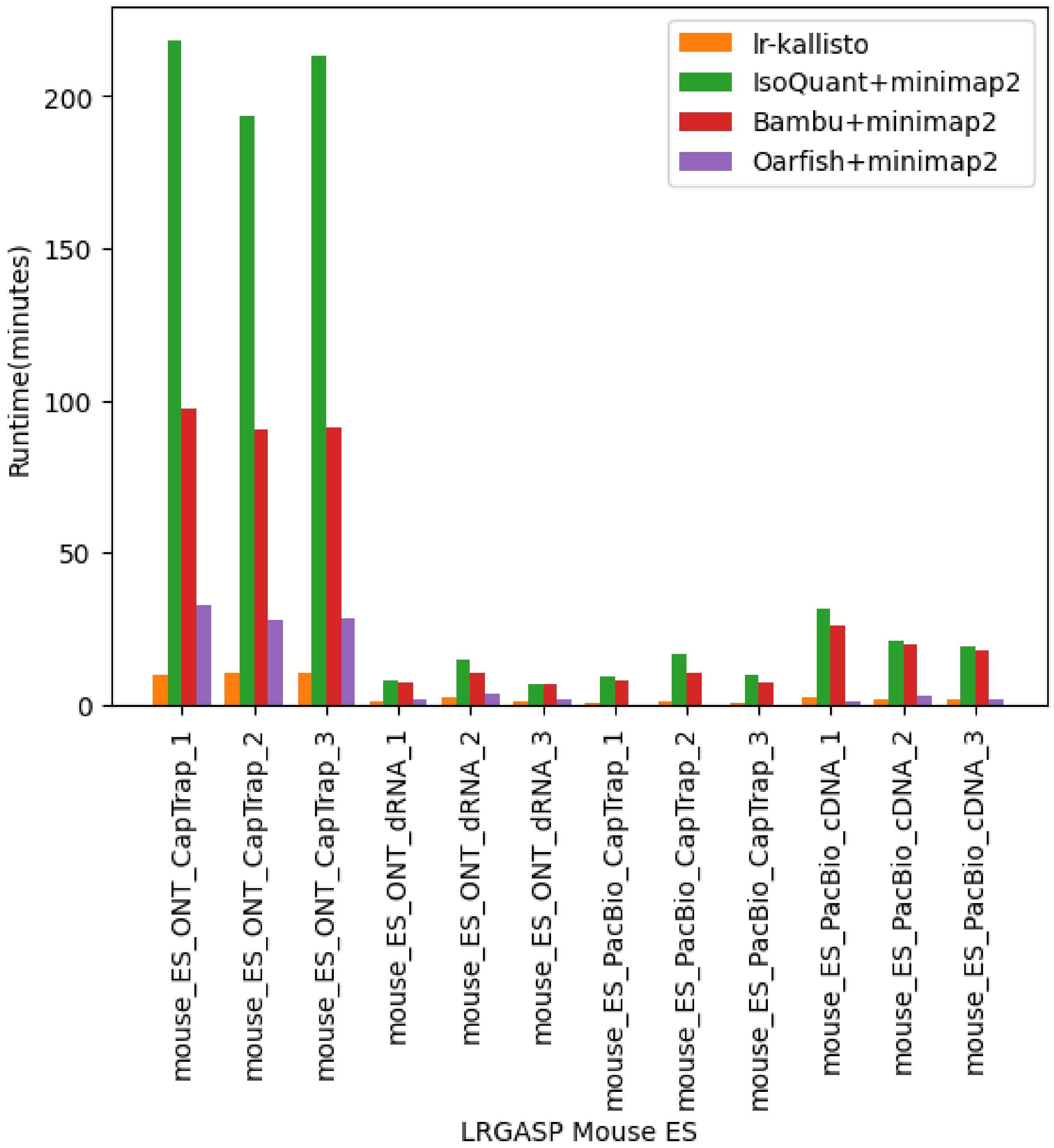

Supplementary Fig. 1c: Runtime performance comparisons for lr-kallisto, IsoQuant, Bambu and Oarfish.

I. ONT vs Illumina

i.

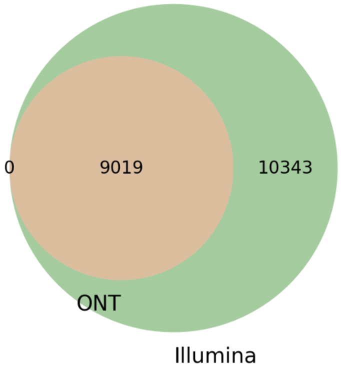

ii.

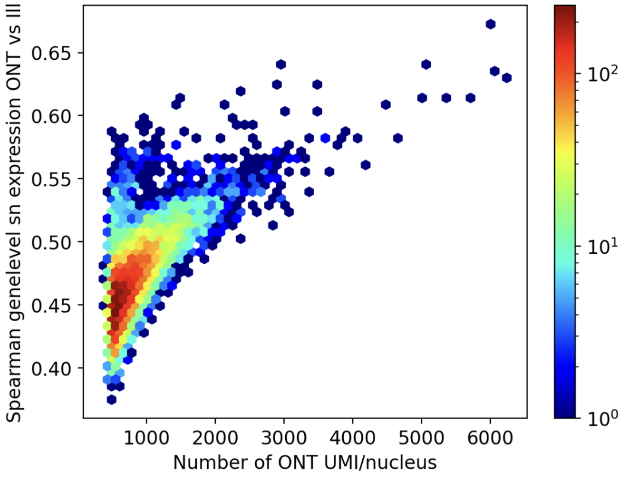

II. Illumina, randO vs polyT

i.

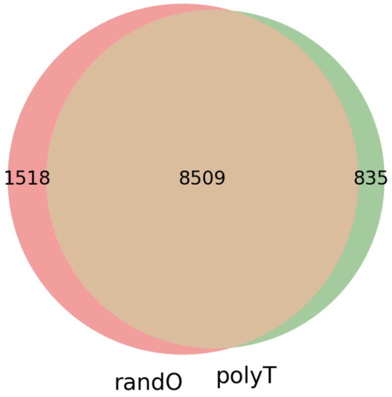

ii.

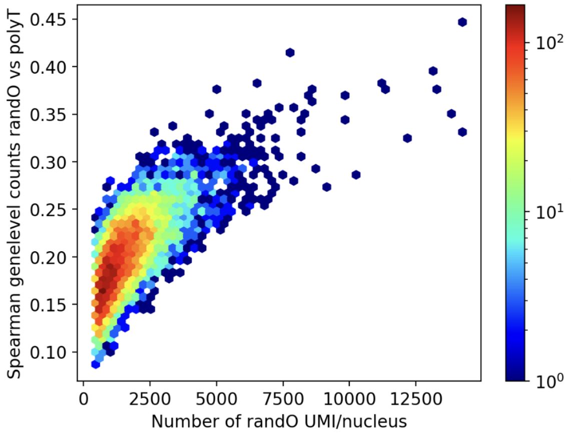

III. ONT, randO vs polyT

i.

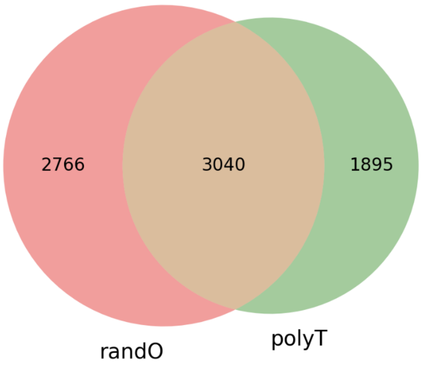

ii.

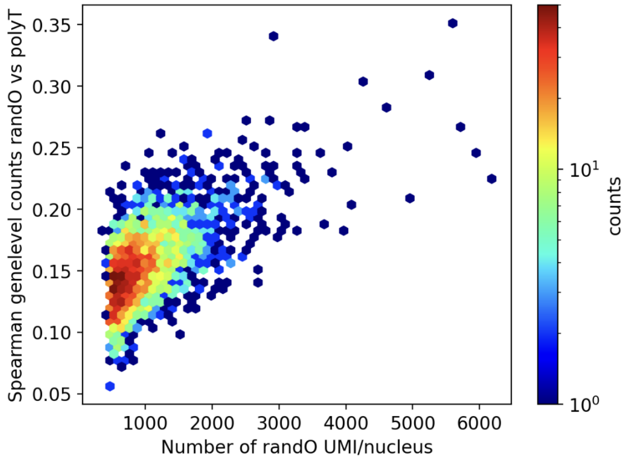

Supplementary Fig. 1d: I. i. Venn diagram of barcodes in ONT and Illumina. ii. Number of ONT UMI/nucleus vs Spearman correlation between ONT and Illumina single-nucleus gene level counts. II. i. Venn diagram of barcodes in Illumina random oligo (randO) and Illumina poly dT. ii. Number of randO UMI/nucleus vs Spearman correlation between Illumina randO and Illumina polydT single-nucleus gene level counts. III. i. Venn diagram of barcodes in ONT random oligo (randO) and ONT poly dT. ii. Number of randO UMI/nucleus vs Spearman correlation between ONT randO and ONT polydT single-nucleus gene level counts.

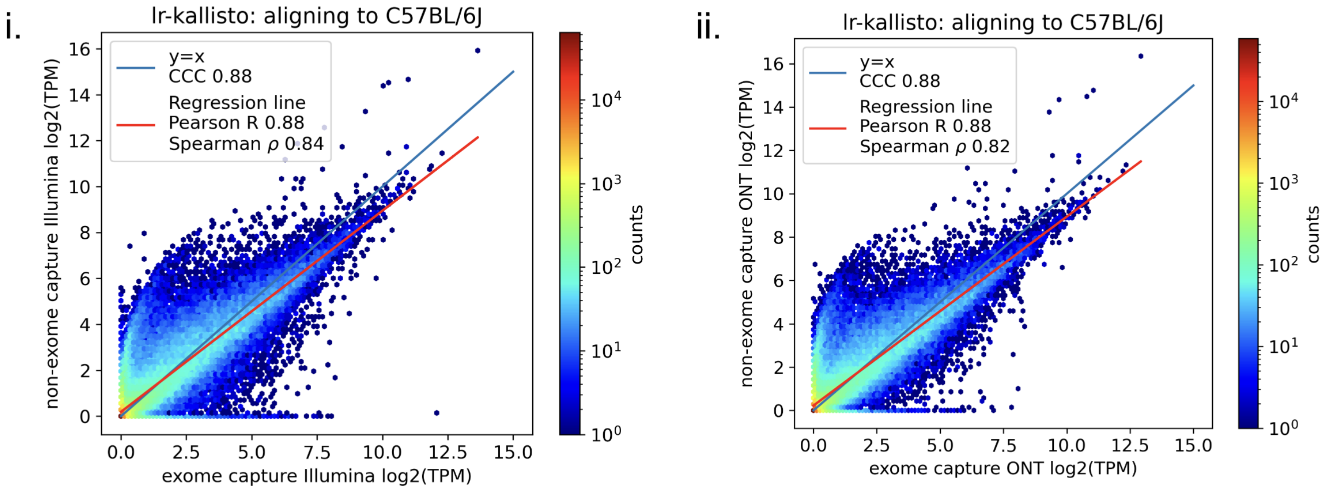

Supplementary Fig. 1e: Contrast of non-exome vs exome capture in Illumina and ONT.

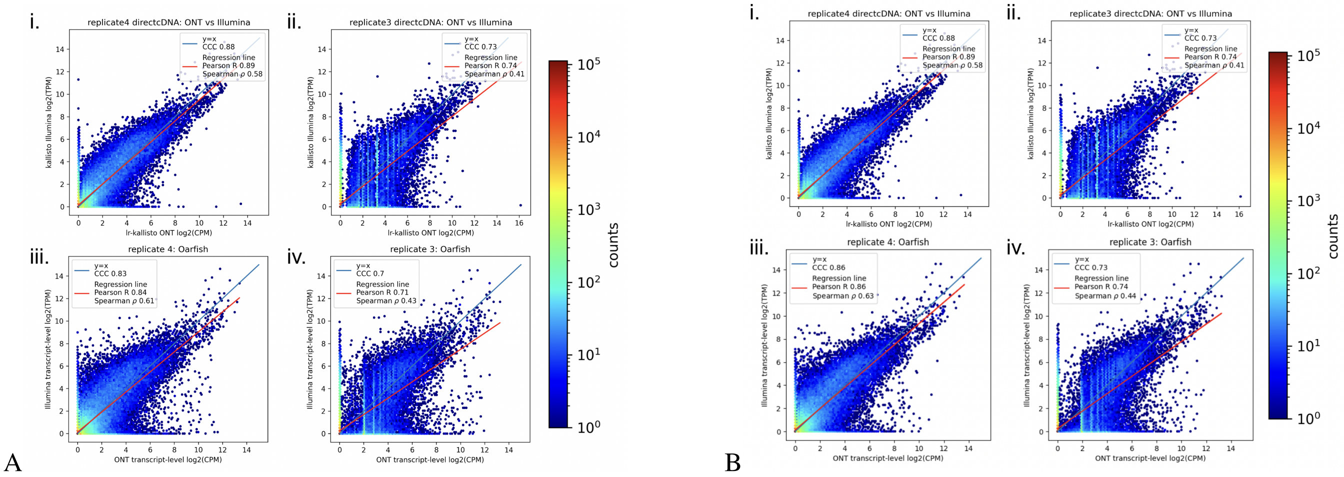

Supplementary Fig. 2a: Performance of lr-kallisto on ONT sequenced direct cDNA libraries from the HCT116 cell line, where panel A is with Oarfish v0.3.1 and panel B is with Oarfish v0.5.1.

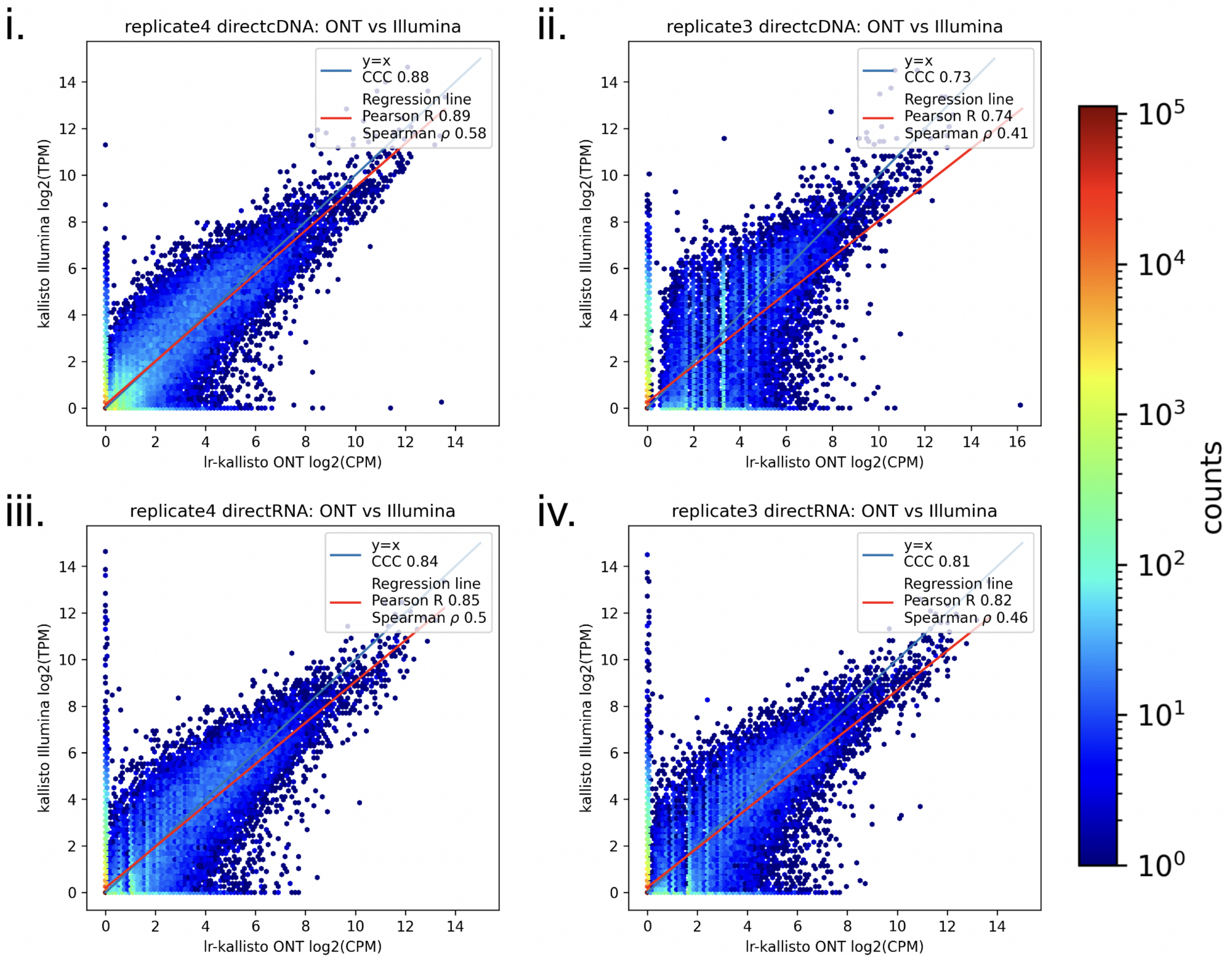

Supplementary Fig. 2b: Comparison of lr-kallisto on ONT sequenced HCT116 cell line libraries generated with directRNA and direct cDNA between two replicates in each.

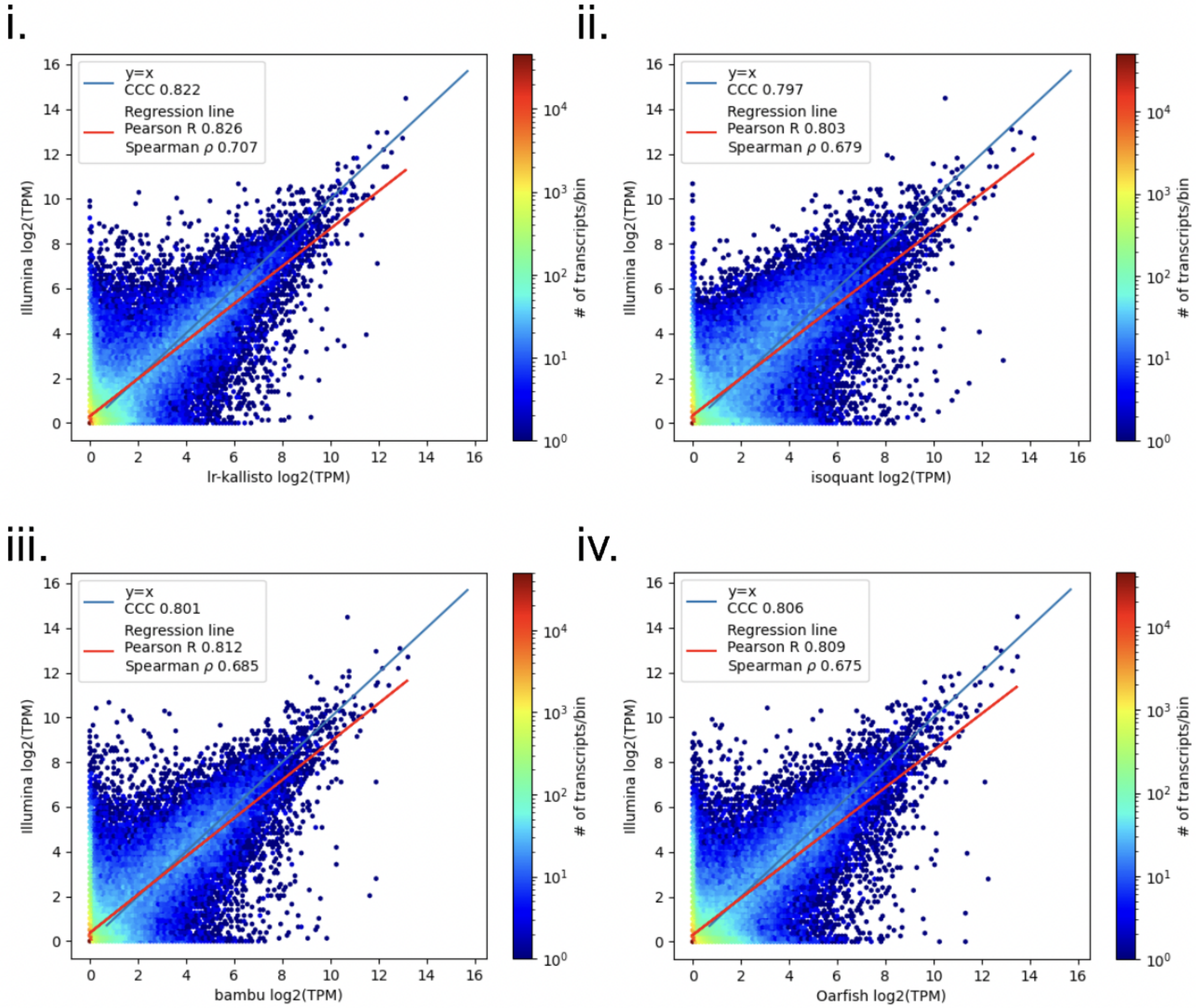

Supplementary Fig. 2c: Evaluation of Bambu, IsoQuant, lr-kallisto, and Oarfish on mouse cortex high-depth PacBio data.

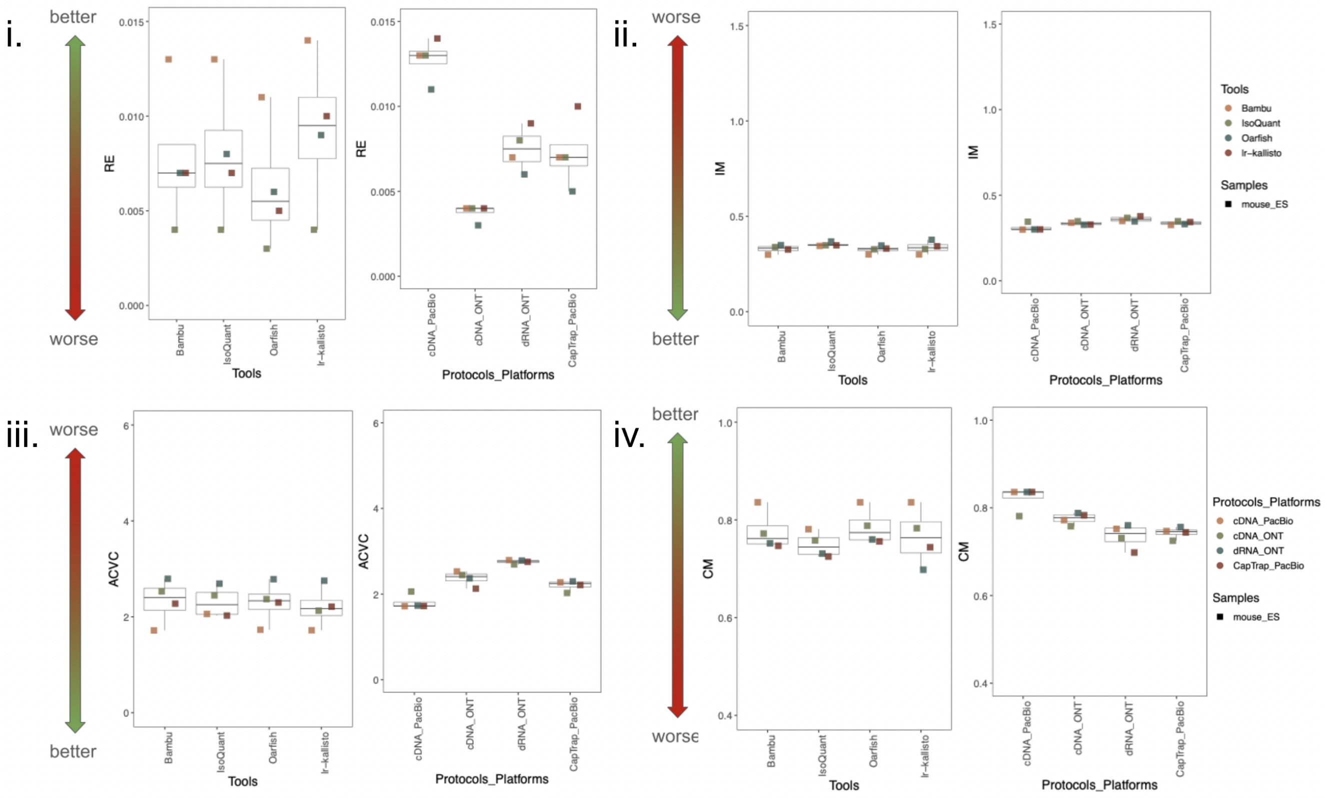

Supplementary Fig. 3: Evaluation of lr-kallisto, Bambu, IsoQuant and Oarfish according to LRGASP challenge 2 metrics in Mouse ES cells.

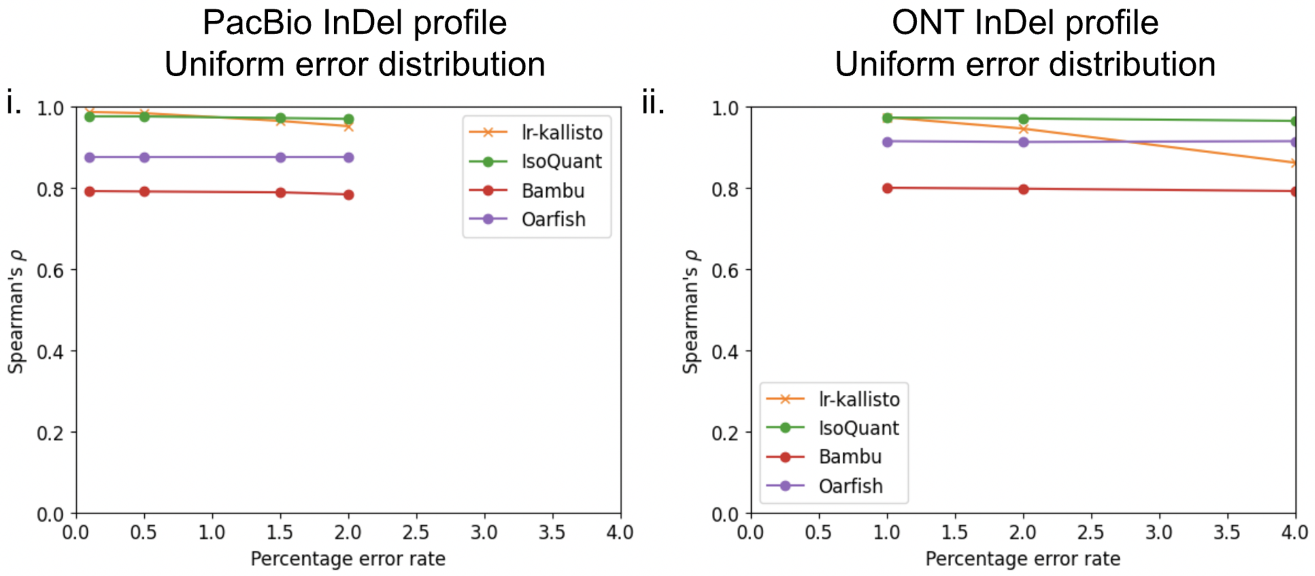

Supplementary Fig. 4a: Benchmarks of Bambu, IsoQuant, lr-kallisto, and Oarfish on simulations with a range of error parameters.

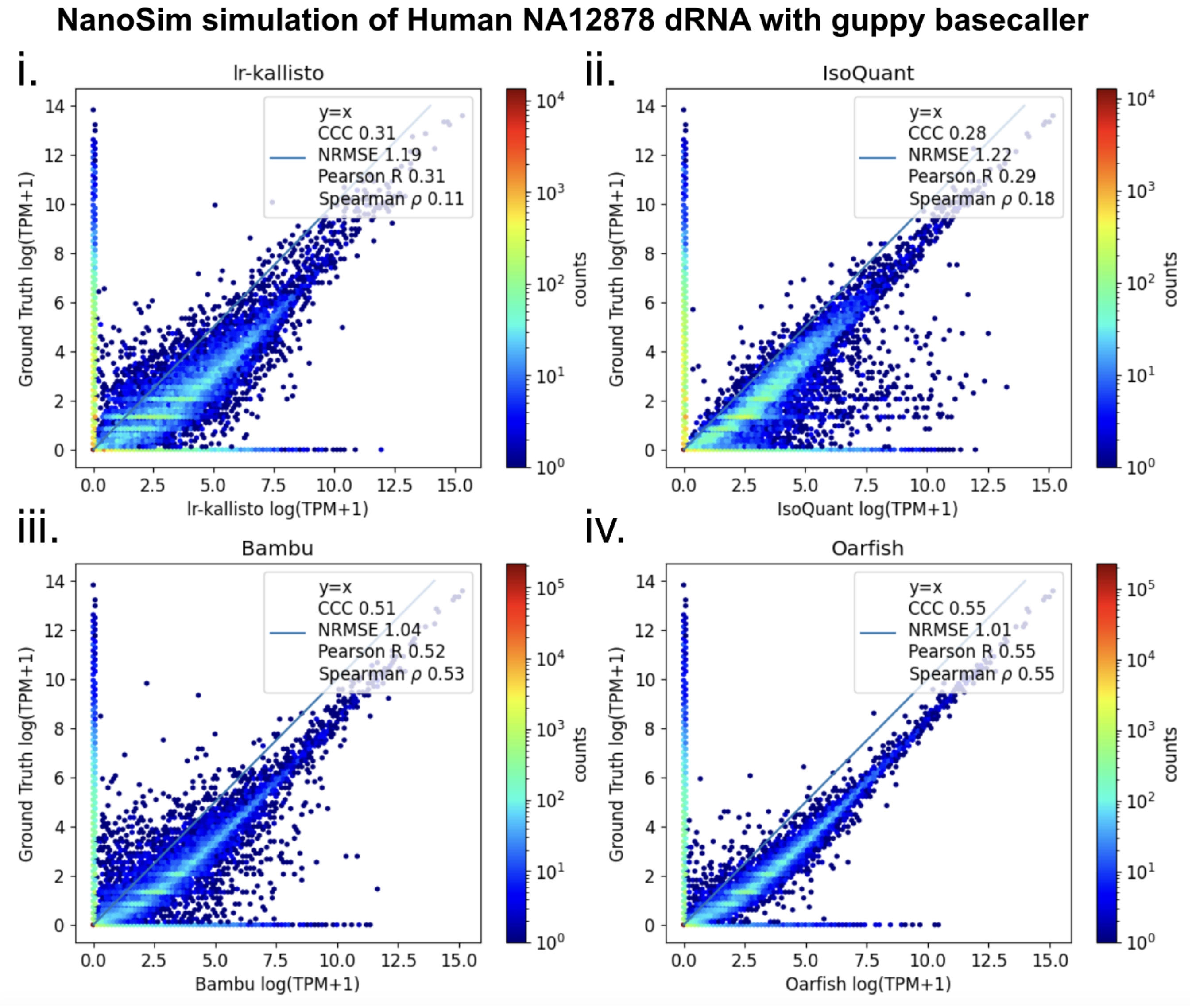

Supplementary Fig. 4b: Performance on all annotated transcripts at ONT 11.2% sequencing error rate.

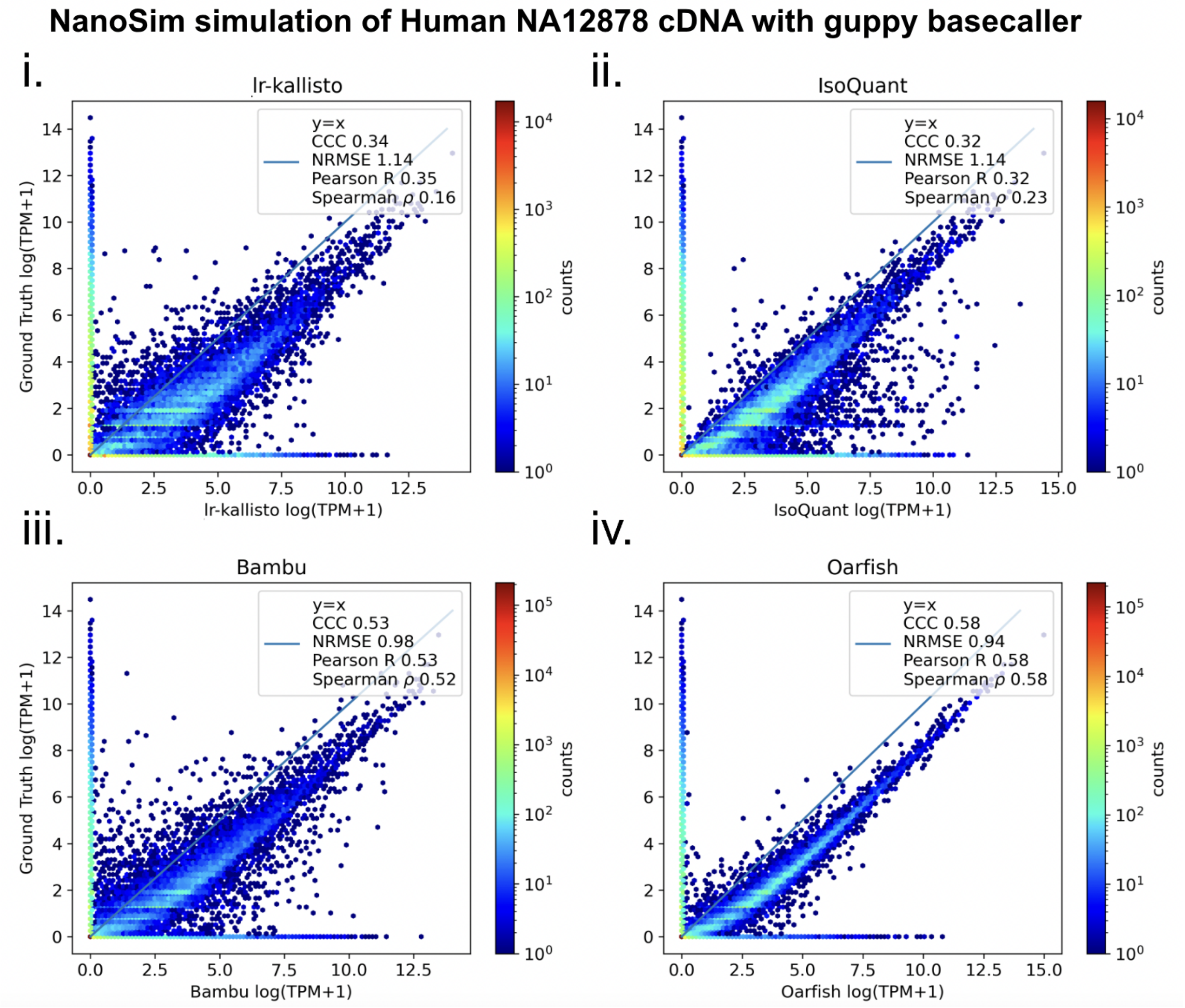

Supplementary Fig. 4c: Performance on all annotated transcripts at ONT 15.2% sequencing error rate.

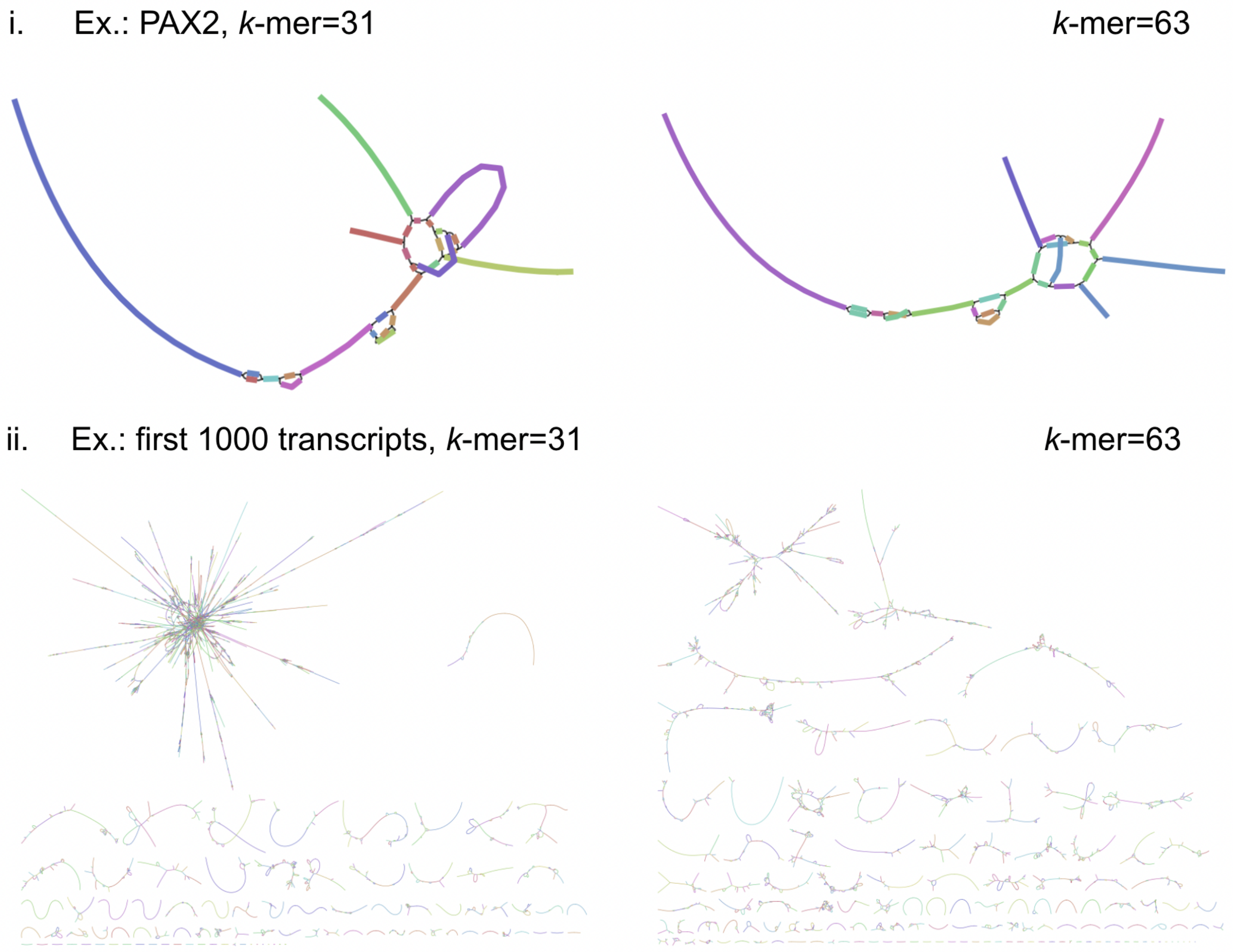

Supplementary Fig. 5: lr-kallisto transcript de Bruijn Graph bandage plots.
