# Supplementary material for: Long-read sequencing transcriptome quantification with lr-kallisto
Source: bioRxiv. 2024 Jul 19:2024.07.19.604364. Preprint. [Version 1] doi: 10.1101/2024.07.19.604364 (PMC11275803; doi:10.1101/2024.07.19.604364)
Supplement: Supplementary file 1 [file media-1.pdf]

Supplementary Figures
Long-read sequencing transcriptome quantification with lr-kallisto

Rebekah Loving[1], Delaney K. Sullivan[1,2], Fairlie Reese[3,4], Elisabeth Rebboah[3,4], Jasmine Sakr[3,4], Narges Rezaie[3,4], Heidi Y. Liang[3,4], Ghassan Filimban[3,4], Shimako Kawauchi[3], Conrad Oakes[1], Diane Trout[1], Brian A. Williams[1], Grant MacGregor[3], Barbara J. Wold[1], Ali Mortazavi[3,4], and Lior Pachter[1,5]

[1]Division of Biology and Biological Engineering, California Institute of Technology, Pasadena, CA 91125
[2]UCLA-Caltech Medical Scientist Training Program, David Geffen School of Medicine, University of California, Los Angeles, Los Angeles, CA, 90095, USA
[3]Developmental and Cell Biology, University of California Irvine, Irvine, USA
[4]Center for Complex Biological Systems, University of California Irvine, Irvine, USA
[5]Department of Computing and Mathematical Sciences, California Institute of Technology, Pasadena, CA 91125

Correspondence: ***Ali Mortazavi (ali.mortazavi@uci.edu) and Lior Pachter (lpachter@caltech.edu)***

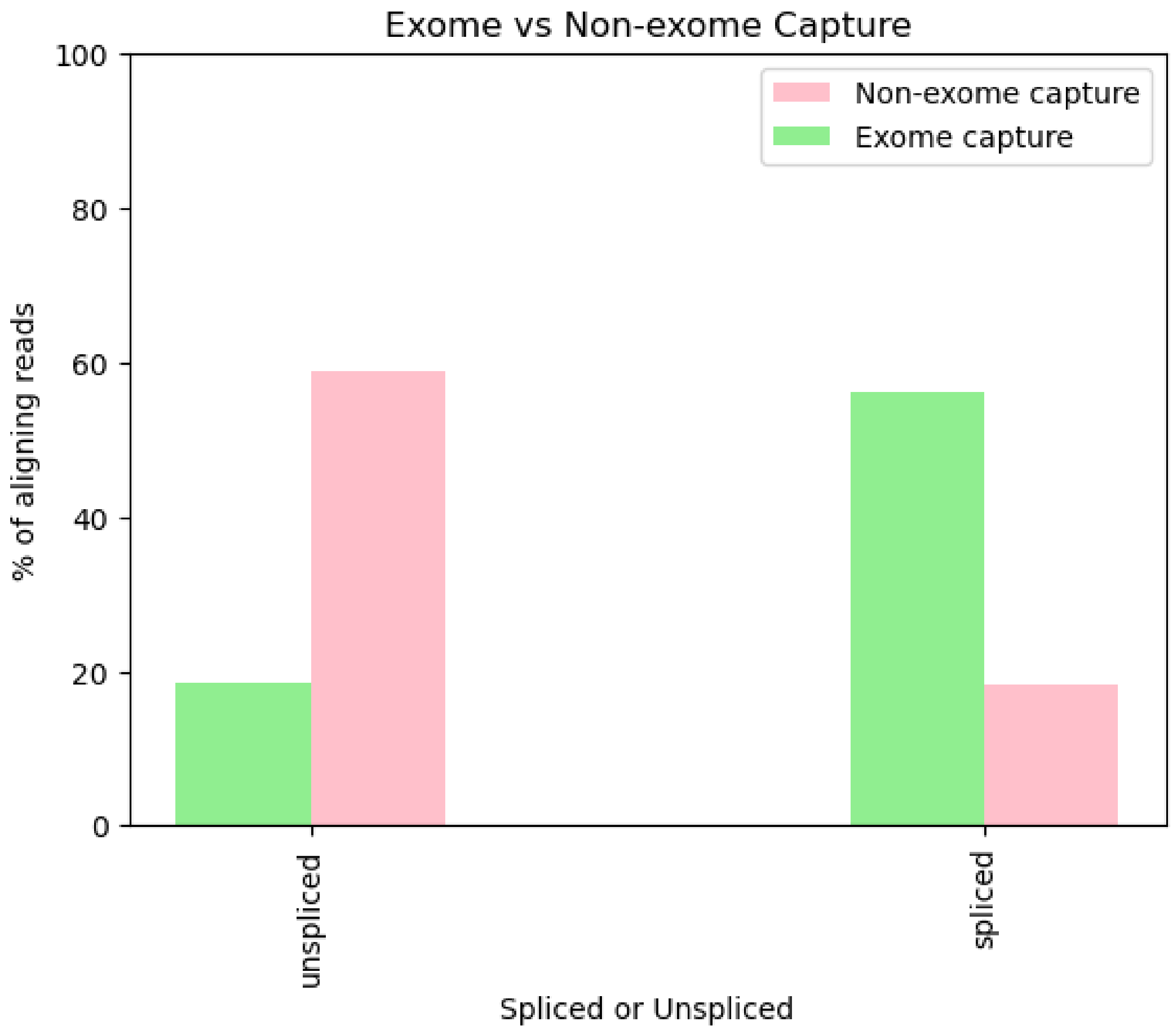

Supplementary Fig. 1a: Comparison of the percent of reads mapping as spliced vs. unspliced reads with and without exome capture.

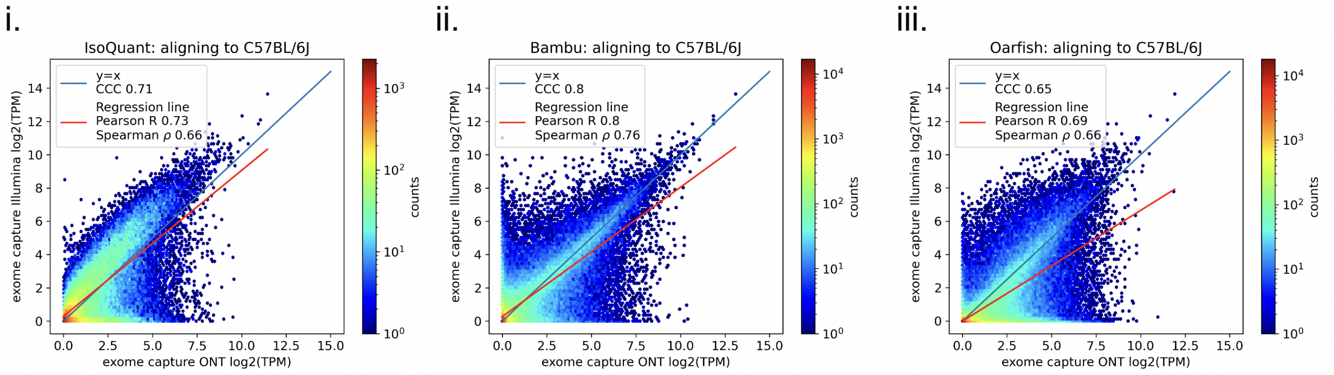

Supplementary Fig. 1b: Quantifications of the C57BL/6J exome capture samples with Bambu, IsoQuant, and Oarfish.

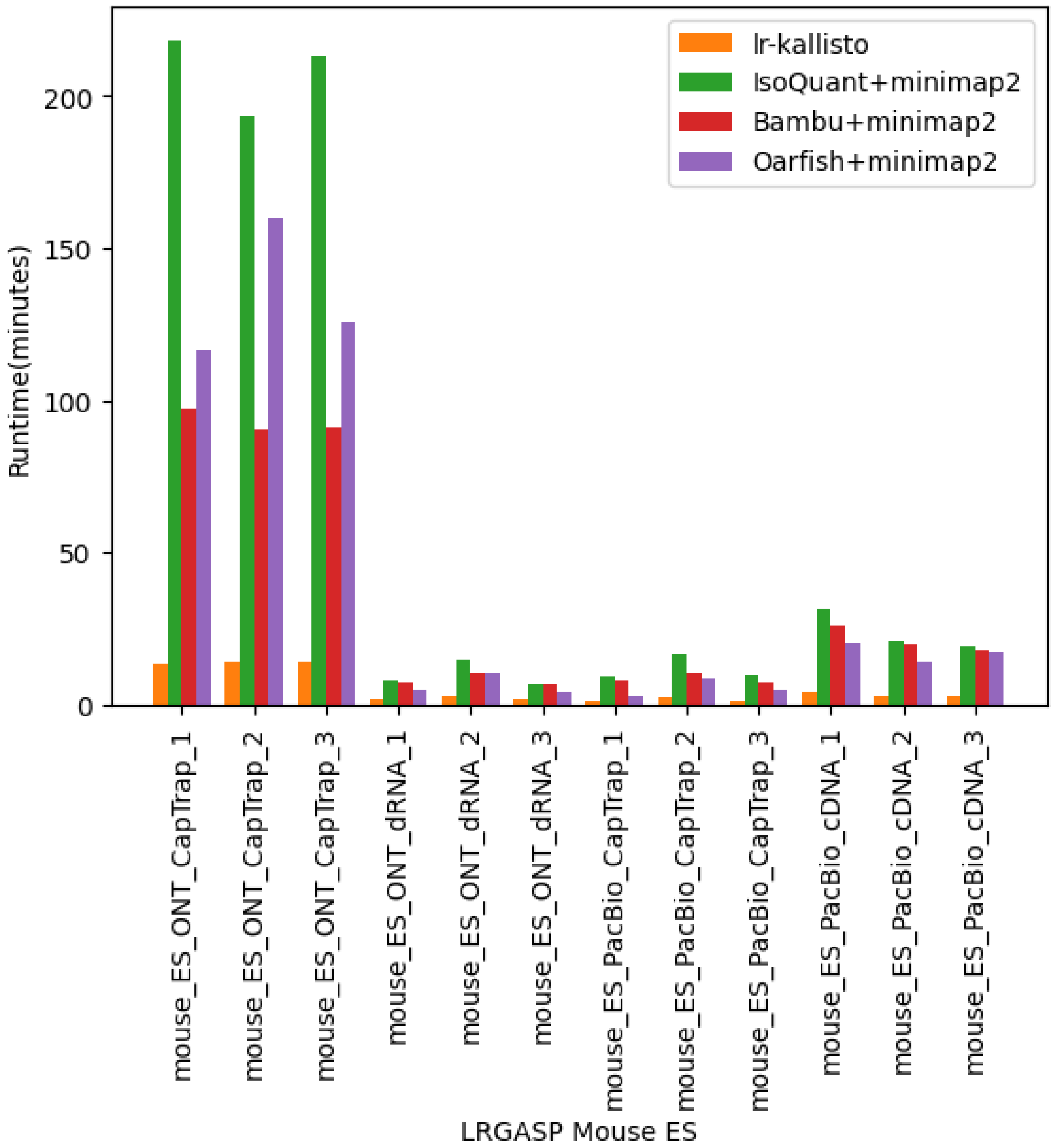

Supplementary Fig. 1c: Runtime performance comparisons for lr-kallisto, IsoQuant, Bambu and Oarfish.

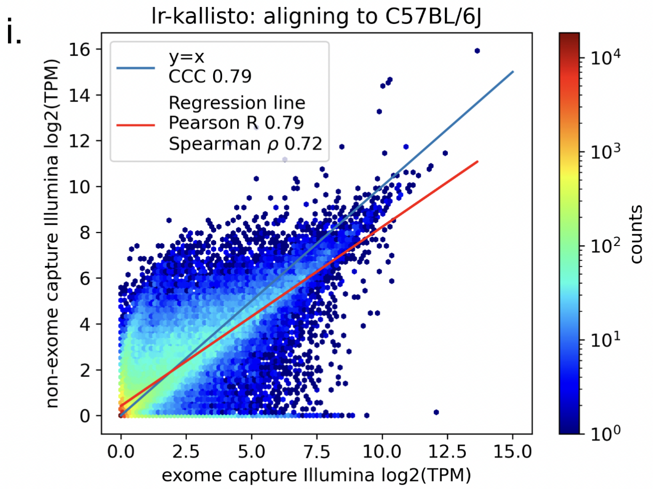

Supplementary Fig. 1d: Contrast of non-exome vs exome capture in Illumina and ONT.

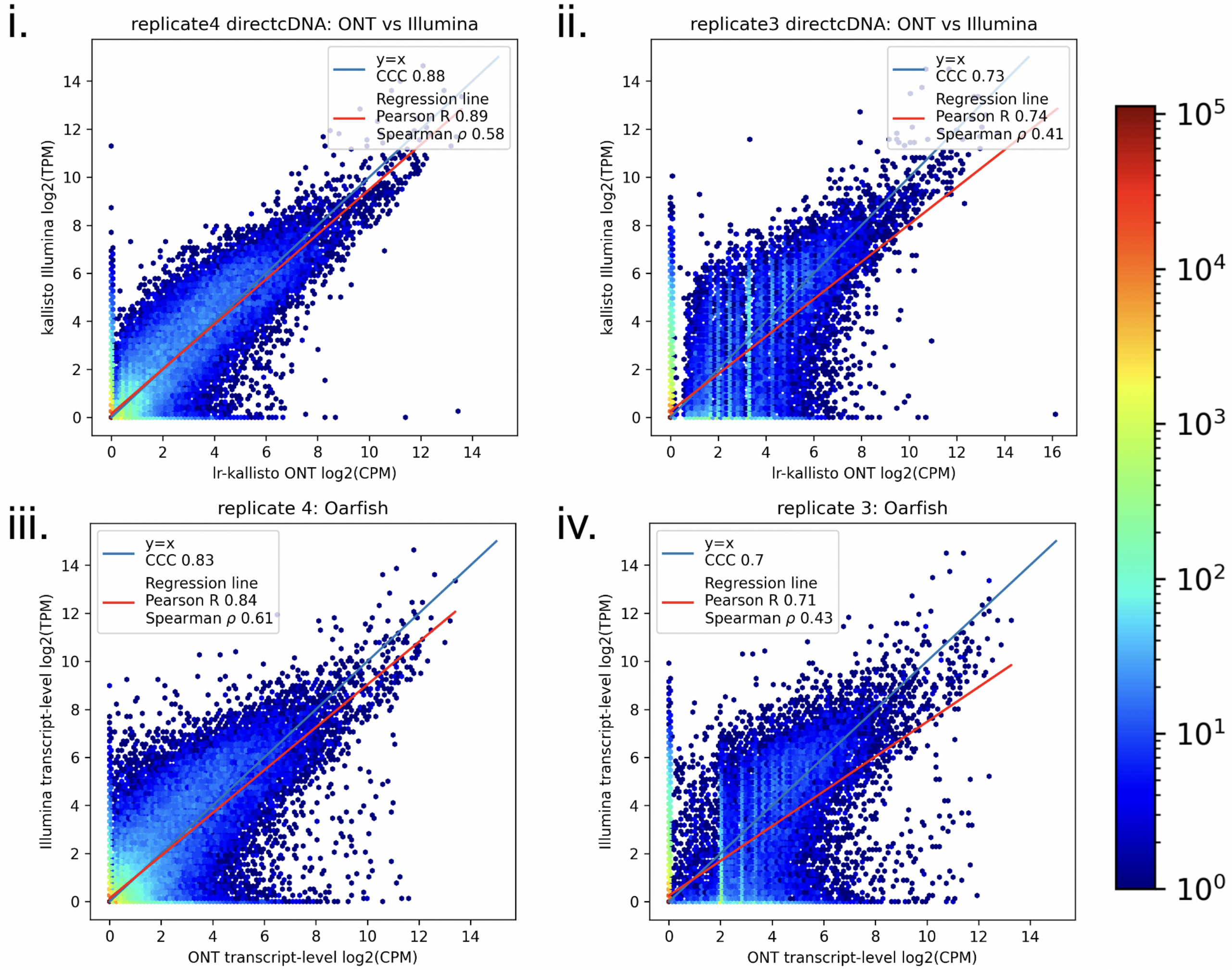

Supplementary Fig. 2a: Performance of lr-kallisto on ONT sequenced direct cDNA libraries from the HCT116 cell line.

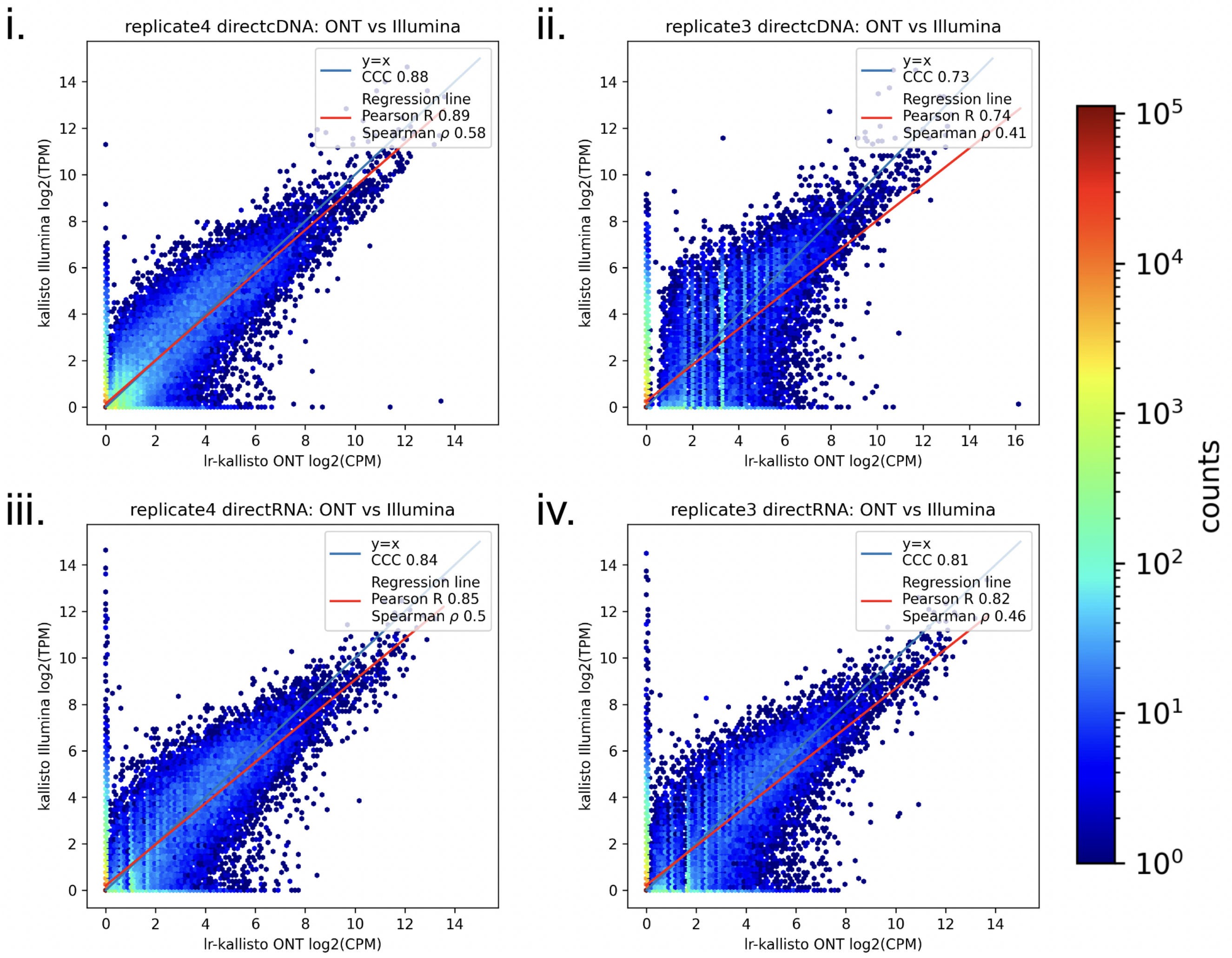

Supplementary Fig. 2b: Comparison of lr-kallisto to Oarfish on ONT sequenced HCT116 cell line libraries generated with directRNA.

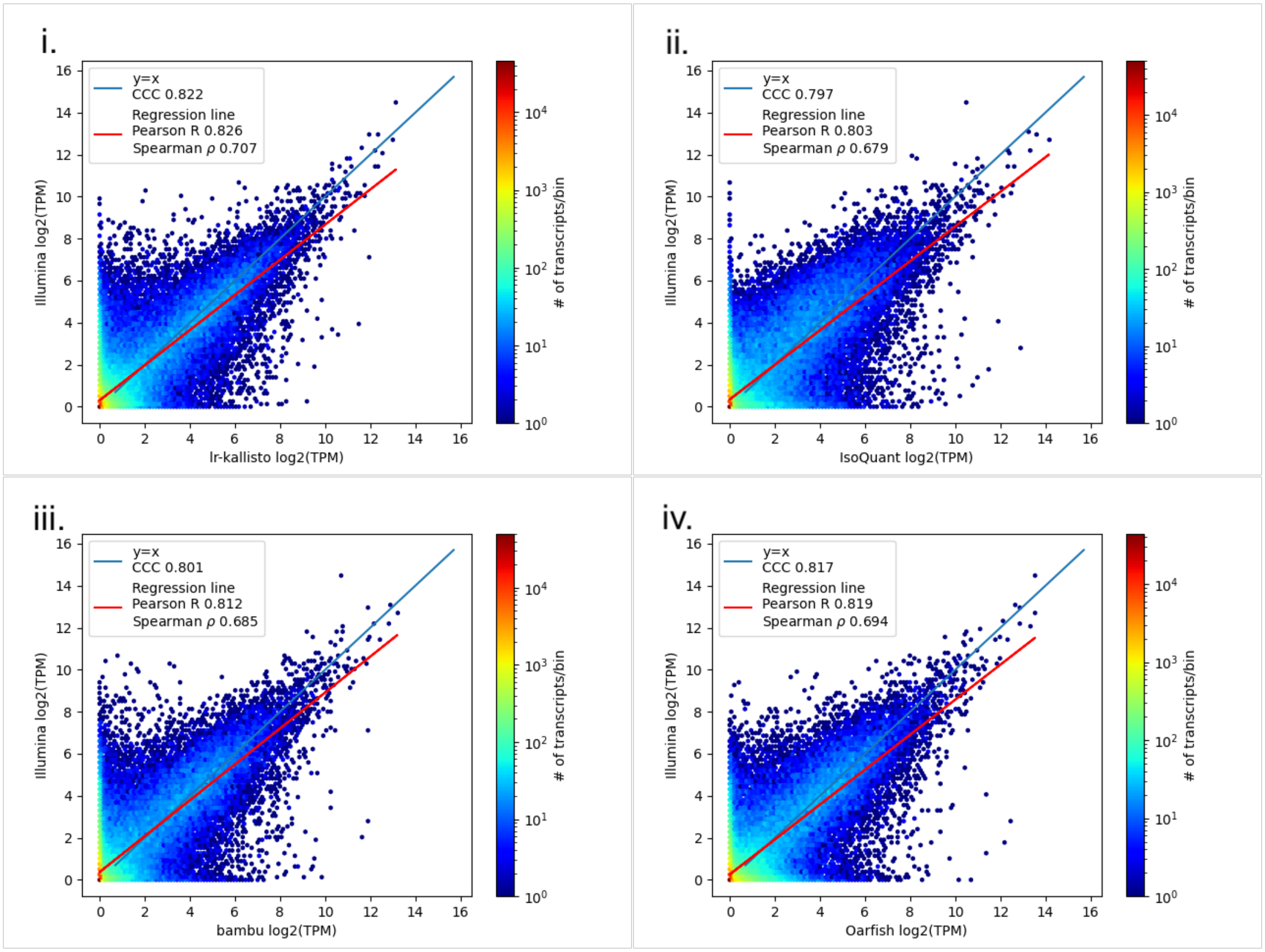

Supplementary Fig. 2c: Evaluation of Bambu, IsoQuant, lr-kallisto, and Oarfish on mouse cortex high-depth PacBio data.

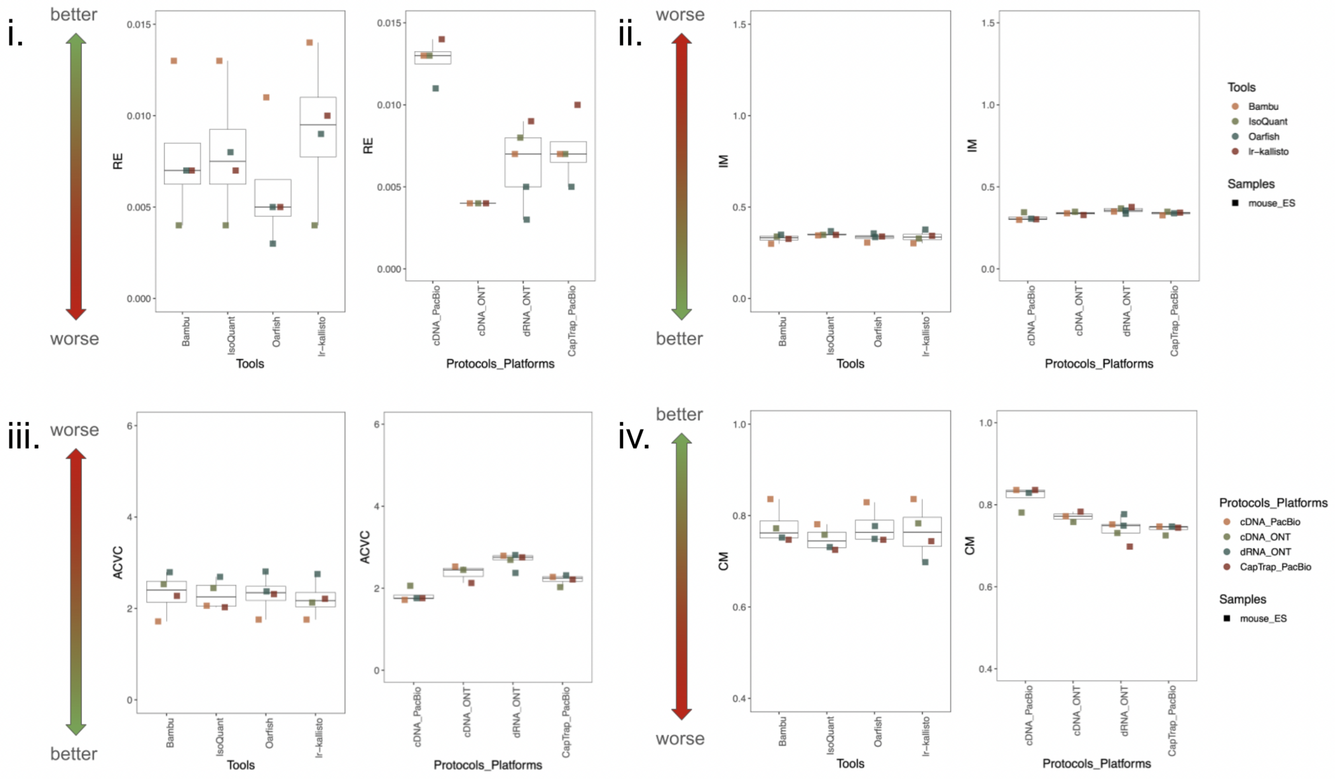

Supplementary Fig. 3: Evaluation of lr-kallisto, Bambu, IsoQuant and Oarfish according to LRGASP challenge 2 metrics in Mouse ES cells.

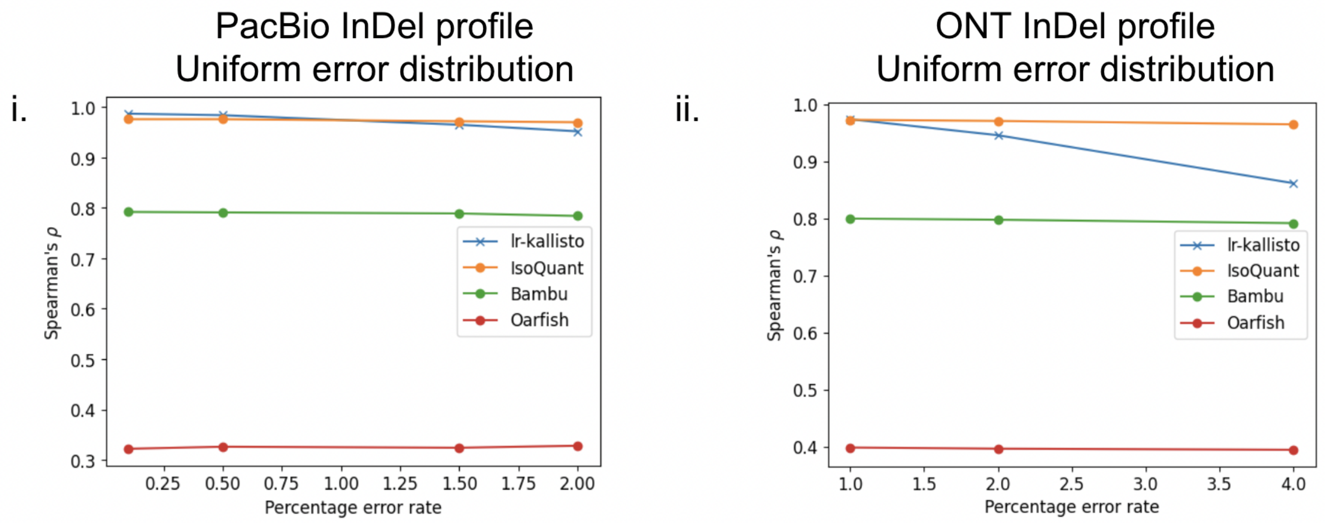

Supplementary Fig. 4a: Benchmarks of Bambu, IsoQuant, lr-kallisto, and Oarfish on simulations with a range of error parameters.

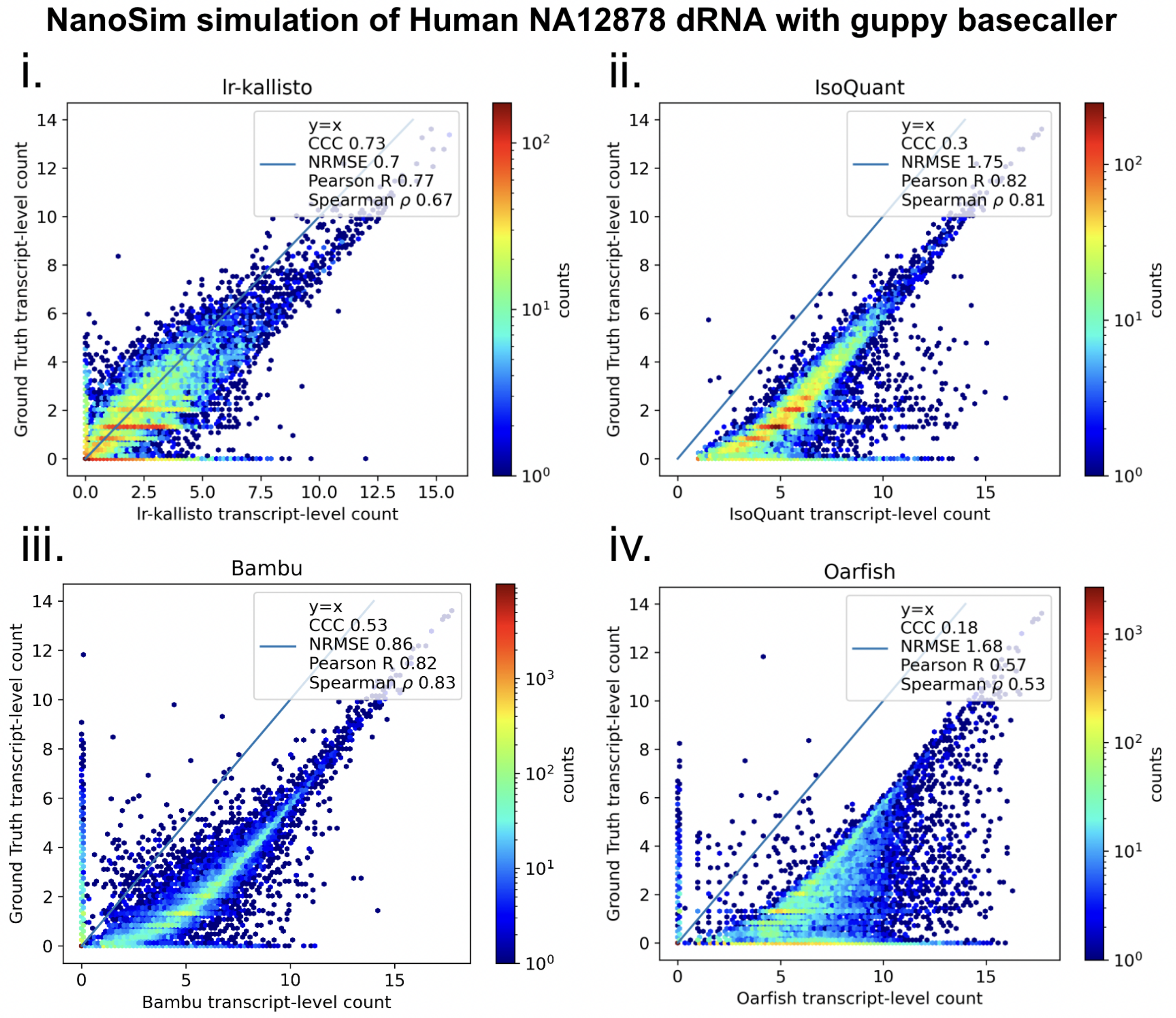

Supplementary Fig. 4b: Performance on detected transcripts at ONT 11.2% sequencing error rate.

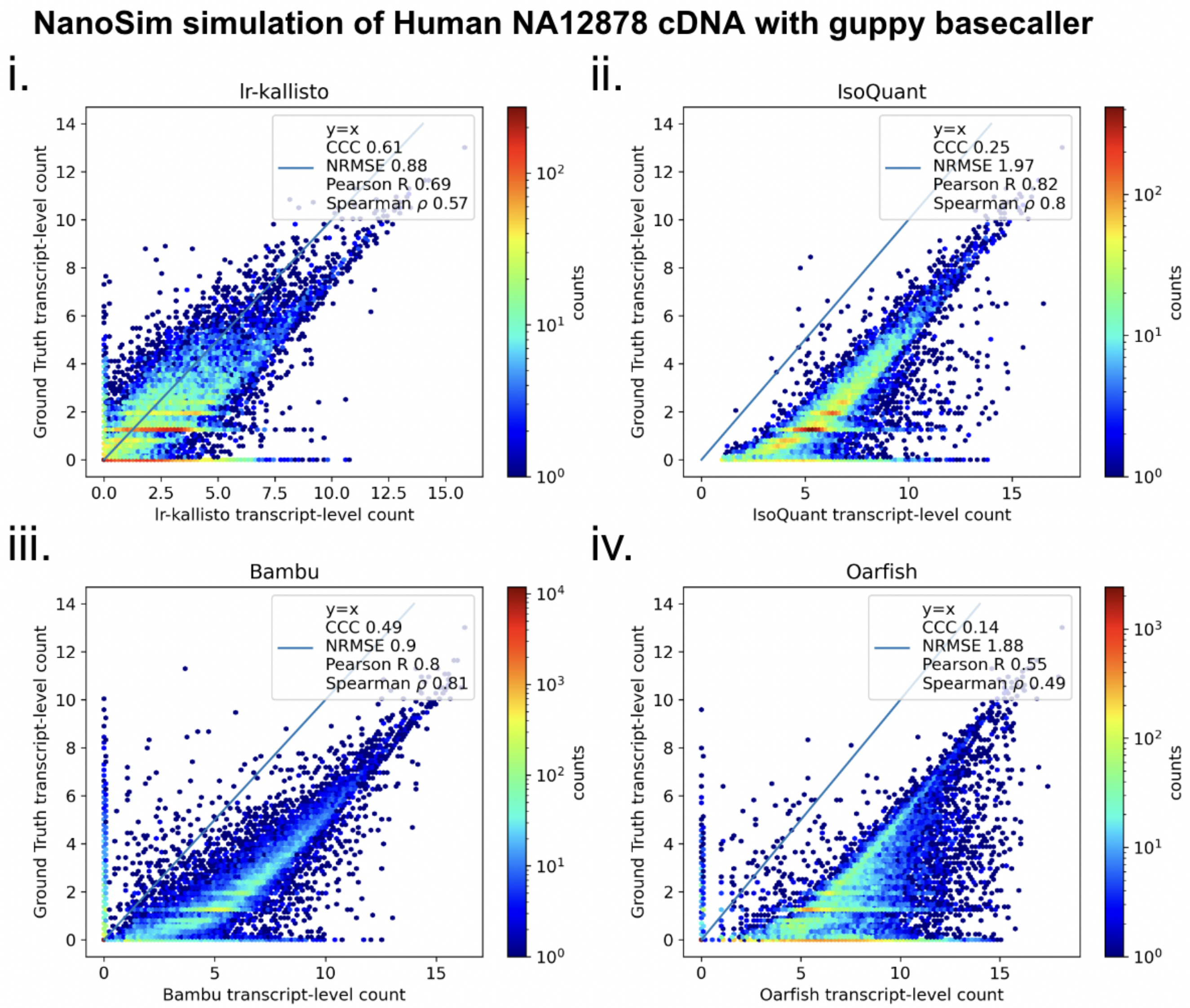

Supplementary Fig. 4c: Performance on detected transcripts at ONT 15.2% sequencing error rate.

NanoSim simulation of Human NA12878 dRNA with guppy basecaller

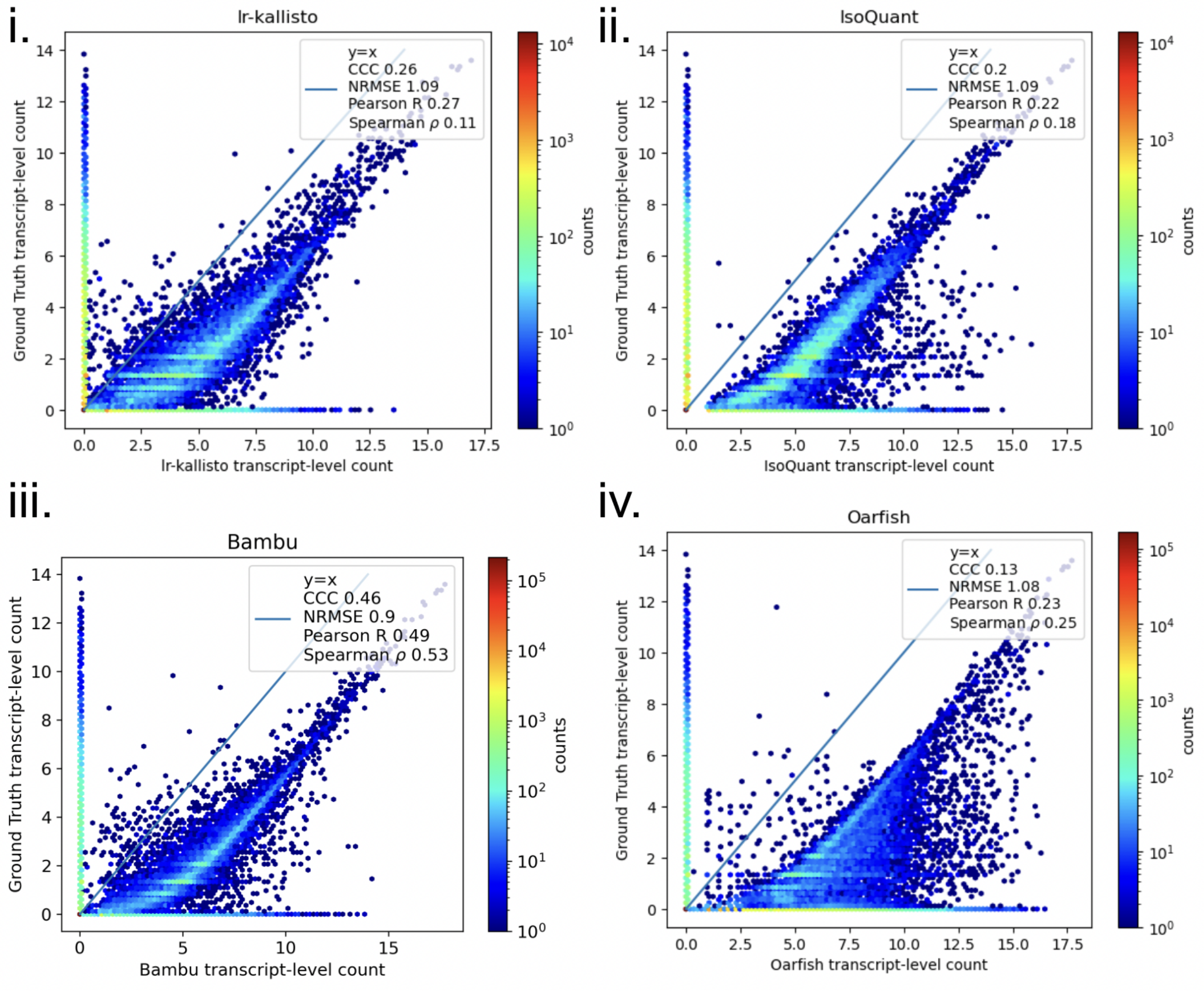

Supplementary Fig. 4d: Performance on all annotated transcripts at ONT 11.2% sequencing error rate.

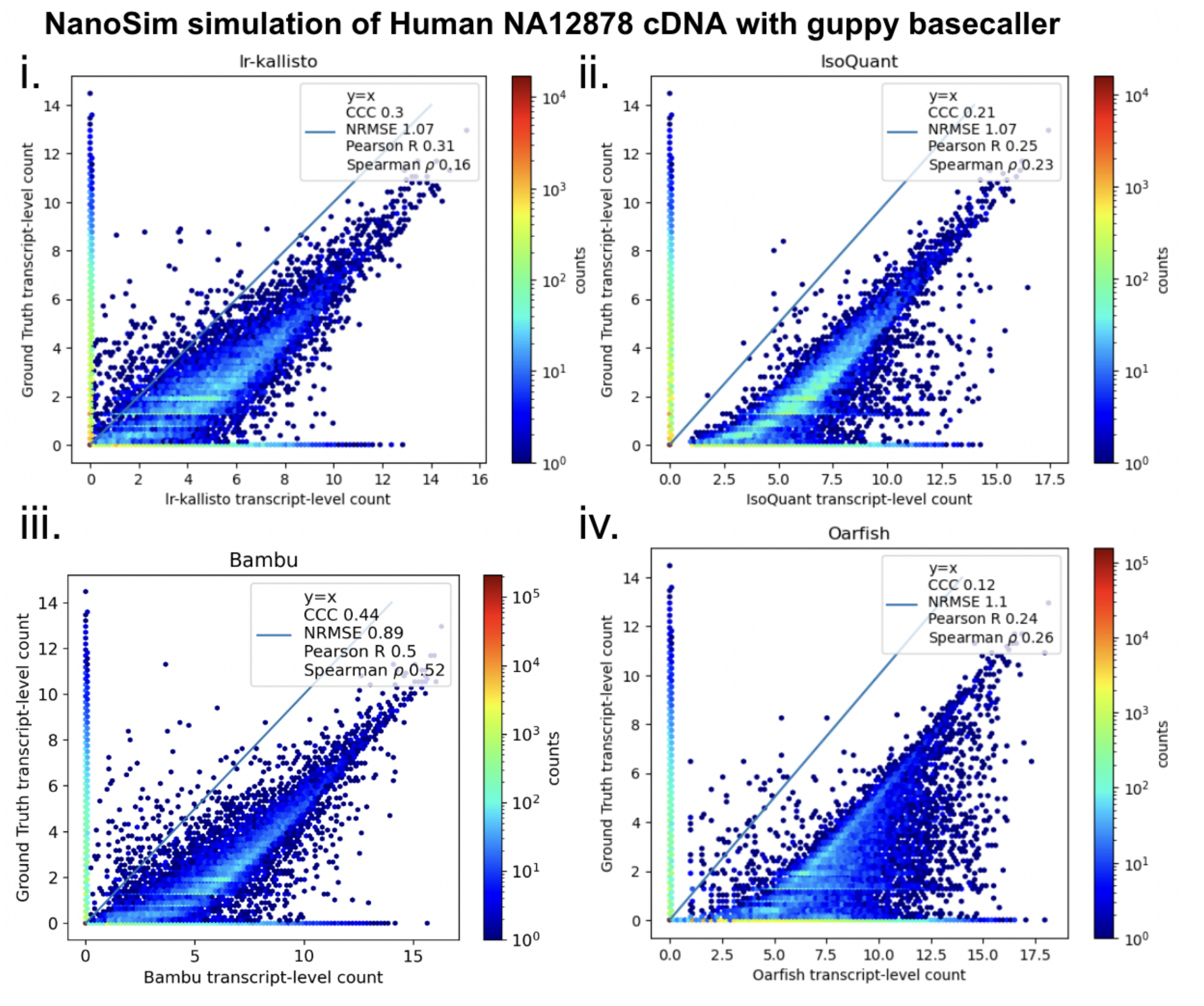

Supplementary Fig. 4e: Performance on all annotated transcripts at ONT 15.2% sequencing error rate.

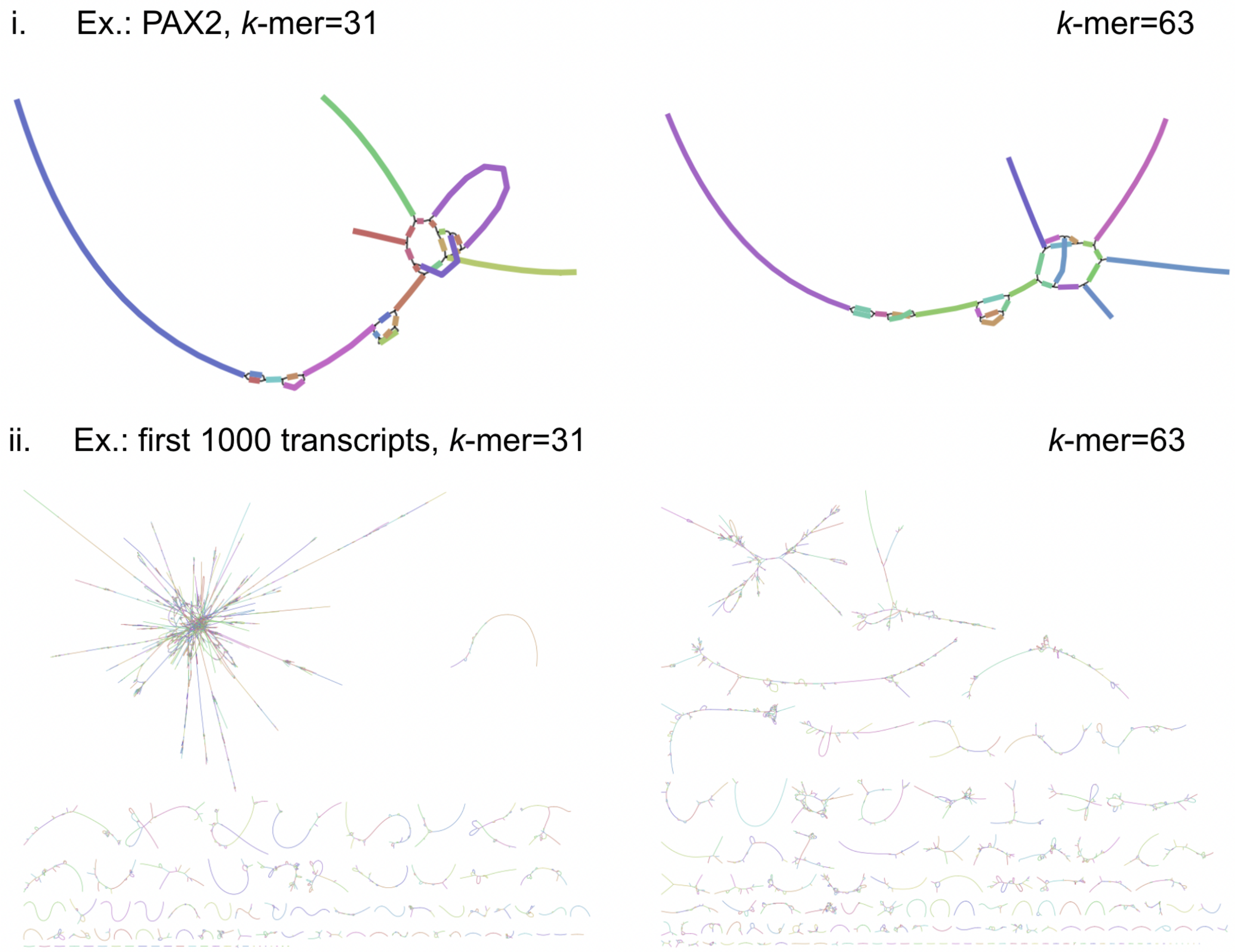

Supplementary Fig. 5: lr-kallisto transcript de Bruijn Graph bandage plots.
